# Predictors of oedema among children hospitalized with severe acute malnutrition in Jimma University Hospital, Ethiopia: a cross sectional study

**DOI:** 10.1186/1471-2431-13-204

**Published:** 2013-12-06

**Authors:** Tsinuel Girma, Pernille Kæstel, Christian Mølgaard, Kim F Michaelsen, Anne-Louise Hother, Henrik Friis

**Affiliations:** 1 Department of Pediatrics and Child Health, Jimma University Specialized Hospital, Jimma, Ethiopia; 2 Department of Nutrition, Exercise and Sports University of Copenhagen Frederiksberg Campus, Rolighedsvej 30, Frederiksberg C DK-1958, Denmark

**Keywords:** Severe acute malnutrition, Oedema, Infection, Risk, Predictors

## Abstract

**Background:**

Severe acute malnutrition has two main clinical manifestations, i.e., oedematous and non-oedematous. However, factors associated with oedema are not well established.

**Methods:**

Children 0.5-14 years of age with SAM (MUAC < 11.0 cm or weight-for-height < 70 % of median and/or nutritional oedema) admitted to the nutrition unit were included. Information on infections before and during admission was collected together with anthropometry. Predictors of oedema was analysed separately for younger (< 60 months) and older children (≥ 60 months).

**Results:**

351 children were recruited (median age: 36 months (interquartile range 24 to 60); 43.3% females). Oedema was detected in 61.1%. The prevalence of oedema increased with age, peaked at 37–59 months (75%) and declined thereafter. Infection was more common in the younger group (33% vs. 8.9%, p < 0.001) and in this group children with oedema had less infections (25.2% vs. 45.1%, p = 0.001). In the older group the prevalence of infections was not different between oedematous and non-oedematous children (5.5% v. 14.3%, p = 0.17). In the younger group oedema was less common in children with TB (OR = 0.20, 95% CI: 0.06, 0.70) or diarrhea (OR = 0.40, 95% CI: 0.21, 0.73).

**Conclusions:**

The proportion of oedema in SAM peaked at three to five years of age and a considerable proportion was above 5 years. Furthermore, the prevalence of infection seemed to be lower among children with oedema. Further studies are needed to better understand the role of infection-immunity interaction.

## Background

Millions of children living in low-income countries suffer from undernutrition; undernutrition contributes to one-third of the deaths in young children [1,2]. Severe acute malnutrition (SAM) affects an estimated 20 million children under 5 years of age [3]. Despite recent improvement in the protocols for treatment of SAM, case-fatality rates of 20-30% are still seen and are higher for oedematous malnutrition [4].

There are two main clinical manifestations of SAM, i.e. oedematous and non-oedematous [5]. However, which factors lead to oedema and the mechanisms behind have been discussed extensively, but remains unknown. In earlier works, oedema in severe malnutrition was explained by dietary protein deficiency [6], and subsequently free-radical-mediated cellular injury was suggested as a mechanism [7]. Recently, researchers suggested a developmental origin, based on a finding in a retrospective observational study [8].

Studies of predictors of SAM are scarce but important to understand the disease process. Existing published works investigated risk factors for undernutrition in general, and mainly in children under the age of five years [9-13]. Large family size, poor maternal nutrition, poverty and unhygienic environment were shown to be risk factors in these studies. Regarding age and gender, however, the results were conflicting.

The aim of this study, therefore, was to identify predictors of oedema among children hospitalized with SAM in the Nutritional Rehabilitation Unit (NRU) of Jimma University Specialized Hospital (JUSH), Ethiopia.

## Methods

### Study setting and subjects

JUSH is located in Jimma Zone in southwest Ethiopia. It has a Paediatric Ward incorporating the NRU, and has been implementing the WHO-based National Guideline for Treatment of Severe Malnutrition [14] since 2004. The NRU receives severely wasted or oedematous patients who have no associated severe acute illness such as severe pneumonia, sepsis, or shock. Severely ill SAM patients are first stabilized in the Critical Care Unit and afterwards transferred to the NRU.

Eligibility for the study required severe wasting (MUAC < 11.0 cm or weight-for-height < 70% of the median of the NCHS growth reference) or nutritional oedema. Children below 6 months of age, those who had life threatening illness, such as shock, and those readmitted with SAM were excluded. Children below 6 months of age were excluded as the diagnosis and treatment of SAM is still not well standardized. Fourteen years was set as the upper age limit since older children were not accepted at the paediatric ward.

### Data collection

Data on household’s water source and toilet facility along with caretaker’s schooling and occupation were obtained by interviewing caretakers, as were history of fever, diarrhea, cough and measles, within one month before admission to hospital. Age of the child was determined from caretakers’ recall. Children were measured naked and body weight recorded to the nearest 10 g using a paediatric scale (Tanita BD 815 MA, Tokyo, Japan). Length was measured in recumbent position for children less than 2 years of age or not able to stand.

Length was recorded to the nearest 0.1 cm using a length board (SECA 416, Hamburg, Germany). When length was measured instead of height in children older than 2 years, 0.5 cm was subtracted from the length. For children older than 2 years, height was measured using a free-standing stadiometer and recoded to the nearest 0.1 cm. MUAC was recorded to the nearest 0.1 cm using a strip (SECA 2012, Hamburg, Germany). Triceps and sub-scapular skin fold thicknesses were measured in duplicates to the nearest 0.2 mm using a Harpenden calliper (Baty International, West Sussex, UK). Presence of pitting oedema was checked by applying a gentle pressure with the thumb for 3–5 seconds. It was registered as “0” if no pitting was detected on the feet. In the presence of pitting, it was recorded as “+” if detected on feet, “++” legs and feet, and “+++” if it included the hands and face.

Infections diagnosed during the admission were taken from the child’s clinical record. The diagnosis of tuberculosis (TB) was based on clinical and radiologic data, according to the Ethiopian National Guideline [15]. Features indicative of TB were chronic symptoms or physical signs suggestive of TB, history of exposure to adult with chronic cough or with TB and suggestive X-rays. For TB suspected children who were able to produce sputum, microscopic sputum examination for acid fast bacilli was done. Tuberculin skin test was unavailable. For febrile patients coming from a malarial area, malaria parasitaemia was examined with Wright stained thick and thin blood films. Pneumonia was diagnosed when a patient had short duration of cough (< 2 weeks) or respiratory difficulty, age-specific fast breathing (above normal for age category), auscultatory and/or chest x-ray findings. Diarrhea was defined as three or more loose stools per day. The clinical case definition for measles was a generalized maculopapular rash lasting for ≥ 3 days, fever (≥ 38.3°C, if measured), and 1 of the following: cough, coryza, or conjunctivitis. Rapid antibody tests were used to diagnose HIV.

Before giving consent, caretakers were given detailed verbal and written information about the study using their language (Afan Oromo). Prior to commencing the study, ethical clearance was granted from the Research Ethical Review Committee, College of Public Health and Medical Sciences, Jimma University. Data were collected by two trained research nurses who spoke the local language. A subset of 20 malnourished children was examined by both nurses to determine percent of technical error of measurement (% TEM). Inter-observer %TEM was 1.1% for MUAC. For biceps, triceps, sub-scapular and suprailiac skinfolds measurements inter-observer %TEM was between 2.6 and 4.8%. Intra-observer %TEM for MUAC was < 0.5% for both nurses, whereas it was between 1.6 and 3.7% for the four skin folds. The study was conducted from December 2009 to October 2011.

### Statistics and data handling

Mean ± standard deviation (SD) median (25^th^; 75^th^ percentile) were used for continuous and percentages for categorical variables when analyzing as well as presenting data. Analysis was done stratified by age, using cut-off 60 months. Chi square, Fisher’s exact test and student *t*-test were used to test for differences in proportion or mean between groups. Simple and multiple logistic regressions were employed to identify predictors of oedema, and odds ratio (OR) with 95% confidence interval (CI) was reported. All the variables except “reported illness” were used in regression; the variable was omitted because of possible overlap in its information with “co-infection”. Data was double entered using EpiData version 3 (EpiData Association, Odense, Denmark). Stata/IC 11.2 (StataCorp, Texas) was used for data analysis and WHO Anthro Plus v 1.0.3 (WHO, Geneva, Switzerland) to calculate Z-score using WHO growth standards. P-value < 0.05 was considered significant.

## Results

During the study period, a total of 527 SAM children (0.5 to 14 years of age) were admitted to the paediatric ward. From these, 176 (33.4%) were excluded, mainly (96.7%) due to critical illness. No differences were found between excluded and studied children when comparing their mean age (1.6 months, 95% CI, -4.2, 7.4), and the proportions of females (38.6% v. 43.3%, p = 0.30), presence of oedema (66.1% v*.* 61.1%, p = 0.26) and proportion of children under the age of five years (75.6% v. 74.4%, p = 0.76).

In total, 351 children were included in the study. The median age was 36 months (interquartile range 24 to 60), and 261 (74.4%) were under the age of five years. The proportion of females was lower among the younger children compared to older (40.2% v. 52.2%, p = 0.05) (Table 1).

**Table 1:** Characteristics and season of admission for children admitted with severe acute malnutrition

	**Age < 5 years**^**a**^	**Age ≥ 5 years**^**a**^	**p-value**
	**n = 261**	**n = 90**	
Female sex	105 (40.2)	47 (52.2)	0.05
Caretaker of child in hospital			< 0.001
Mother	151 (57.8)	30 (33.3)	
Father	90 (34.5)	51 (56.7)	
Relative	20 (7.7)	9 (10.0)	
Caretaker’s occupation			0.03
Farmer	187 (71.6)	76 (83.5)	
Employed	39 (15.0)	5 (5.5)	
Other^b^	35 (13.4)	9 (10.0)	
Caretaker’s schooling			0.34
No schooling	170 (65.0)	63 (70.0)	
Some schooling	91 (35.0)	27 (30.0)	
Toilet facility	238 (91.5)	81 (90.0)	0.61
Safe water supply^c^	158 (60.8)	49 (54.4)	0.34
Admission per season^d^			0.06
Pre-harvest	129 (49.4)	55 (61.1)	
Post-harvest	132 (50.6)	35 (38.9)	

^a^Values are median (25th; 75th percentile) or n (%).

^b^Unemployed, studying or on pension.

^c^Main source of drinking water for family is from pipe, protected spring or well.

^d^Pre-harvest (June -Nov) and post-harvest (Dec-May).

Among the young children, 151 (57.8%) had their mothers as attendants in the hospital (Table 1). In both age groups most children came from farming families, 187 (71.6%) in the younger and 76 (83.5%) in the older age group. There was no difference between the two age groups in parental schooling, household’s access to toilet facility and safe water (Table 1). More children in the older group were admitted during the pre-harvest (June-Nov) season compared to the post-harvest period (Dec-May). However, there was no apparent seasonal variation for the young age group. The seasonal difference in admission between the two age groups was not significant**.**

The mean Z-scores of weight-for-age (WAZ), height-for-age (HAZ) and BMI-for-age (BMIZ) for young children were −3.7 (95% CI: -4.0, -3.5), -3.4 (95% CI: -3.5,-3.1) and −2.4 (95% CI: -2.6,-2.2), respectively (Table 2). The means of these indices of the younger children, as shown in Table 2, were not different from that of the older children. The proportion of infection was significantly higher among the younger children (33% v. 8.9%, p < 0.001) (Table 2). Pneumonia was the leading infection in both groups, with 23.0% and 4.4% affected, respectively.

**Table 2:** Anthropometry, presence of oedema and illnesses among 351 children admitted with severe acute malnutrition by age group

	**Age < 5 years**^**a**^	**Age ≥ 5 years**^**a**^	**p-value**
	**n = 261**	**n = 90**	
Growth indicators			
Weight, kg	8.1 (7.8,8.4)	14.1 (13.3,15.1)	
Height, cm	77.6 (76.4,78.6)	105.7 (102.8,109.3)	
BMI-for-age Z-score	−2.4 (−2.6,-2.2)	−2.6 (−3.1,-2.2)	0.31
MUAC, cm	11.1 (11.0,11.3)	12.0 (11.6,12.3)	< 0.001
Weight-for-age Z-score	−3.7 (−4.0,-3.5)	−3.5 (−3.8,-3.2)	0.27
Height-for-age Z-score	−3.4 (−3.5,-3.1)	−3.0 (−3.3,-2.7)	0.14
Weight-for-height Z-score^b^	−3.6 (1.3)		
MUAC-for-age Z-score^b^	−4.0 (−4.1,-3.8)		
Clinical			
Bilateral pedal pitting oedema	159 (61.0)	55 (61.1)	0.87
HIV status			0.80
Negative	172 (66.3)	61 (67.8)	
Positive	6 (2.3)	3 (3.3)	
Unknown	82 (31.4)	26 (28.9)	
Co-infection ( ≥ 1)^c^	86 (33.0)	8 (8.9)	< 0.001
Pneumonia	60 (23.0)	4 (4.4)	< 0.001
Diarrhea	58 (22.2)	3 (3.3)	< 0.001
Tuberculosis (all forms)	14 (5.4)	3 (3.3)	0.58
Malaria	8 (3.1)	1 (1.1)	0.46
Reported illness ( ≥ 1)^d^	237 (90.8)	81 (90.0)	0.55
Fever	182 (70.0)	69 (65.6)	0.48
Diarrhea	169 (65.0)	56 (62.2)	0.53
Cough or difficult breathing	145 (55.8)	42 (46.7)	0.18
Measles	16 (6.2)	5 (5.6)	0.81

^a^Values are mean (95% confidence interval), mean (± standard deviation) or n (%).

^b^Not possible to calculate z-score for age > 5-years using WHO growth standard.

^c^Major diagnoses during admission.

^d^Reported symptoms or illness within one month before admission.

**Figure 1:**
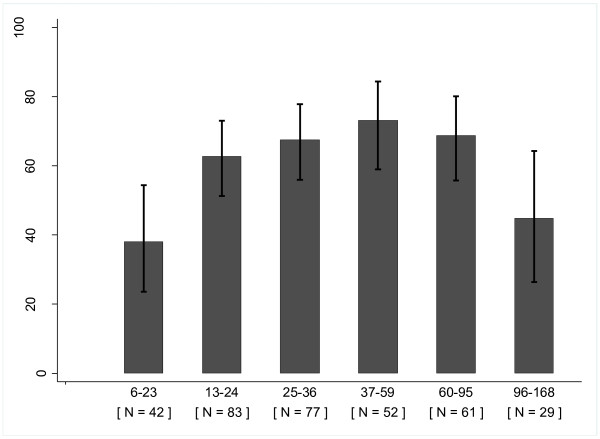
**Percentage of severely malnourished children with oedema by age category in months.** The error bars represent 95% confidence intervals

Oedema was present in 214 (61.1%) children (Table 2). Among these children 102 (47.7%), 59 (27.6%) and 53 (24.8%) had oedema of grade “+”, “++”, and “+++”, respectively (data not shown in table). There was no difference in the proportion and grade of oedema between the two age groups (p = 0.87). In the younger group, the proportion of oedema almost doubled after infancy and peaked at three to five years of age (Figure 1). The proportion of oedema was about one third lower among 96–168 months old children compared to 60–95 months (p = 0.003). However, in both age groups the mean HAZ and admissions seasons were comparable between children with and without oedema (Table 3).

**Table 3:** Age, sex, height, weight, admission season and illness of severely malnourished children by presence of oedema and age group

	**Non-oedematous**^**a**^	**Oedematous**^**a**^	**P-value**
	**n = 102**	**n = 102**	
< 5 yr			
Age category, mo			0.001
6-12	31 (30.4)	17 (10.7)	
13-24	31 (30.4)	52 (32.7)	
25-36	25 (24.5)	52 (32.7)	
37-59	15 (14.7)	38 (24.0)	
Height-for-age Z-score	−3.5 ± 1.8	−3.2 ± 1.6	0.28
Female sex	38 (37.3)	67 (42.1)	0.43
Admission season^b^			
Pre-harvest	56 (55.0)	73 (45.9)	0.21
Post-harvest	46 (45.0)	86 (54.1)	
Co-infection^c^	46 (45.1)	40 (25.2)	0.001
TB	10 (9.8)	4 (2.5)	0.02
Pneumonia	33 (32.4)	27 (17.0)	0.01
Diarrhea	33 (32.4)	25 (15.7)	0.002
Malaria	2 (2.0)	6 (3.8)	0.49
HIV status			0.08
Negative	67 (65.7)	106 (66.7)	
Positive	5 (5.0)	1 (0.6)	
Unknown	30 (29.3)	52 (32.7)	
Reported illness^d^	96 (94.1)	141 (88.7)	0.15
Fever	76 (75.0)	106 (66.7)	0.06
Cough or difficult breathing	69 (67.6)	76 (47.8)	0.001
Diarrhea	63 (61.8)	106 (66.7)	0.52
Measles	9 (8.8)	7 (4.4)	0.20
≥ 5 yr	n = 35	n = 55	
Age category, mo			0.03
60-95	19 (54.3)	42 (76.4)	
96-168	16 (45.7)	13 (23.6)	
Height-for-age Z-score	−3.3 ± 1.5	−3.7 ± 1.2	0.51
Female sex	22 (63.0)	25 (45.5)	0.11
Admission season^b^			
Pre-harvest	21 (60.0)	34 (61.8)	0.86
Post-harvest	14 (40.0)	21 (38.1)	
Co-infection^c^	5 (14.3)	3 (5.5)	0.17
TB	2 (5.7)	1 (1.8)	0.34
Pneumonia	2 (5.7)	2 (11.7)	0.64
Diarrhea	2 (5.7)	1 (5.9)	0.56
Malaria	1 (2.8)	-	0.39
HIV status			0.30
Negative	21 (60.0)	40 (72.7)	
Positive	2 (5.7)	1 (1.8)	
Unknown	12 (34.3)	14 (25.5)	
Reported illness^d^			0.72
Fever	26 (74.3)	33 (60.0)	0.17
Cough or difficult breathing	19 (54.3)	23 (41.8)	0.25
Diarrhea	22 (63.0)	34 (61.8)	0.92
Measles	1 (2.8)	4 (7.3)	0.65

^a^ Values are mean ± standard deviations or n (%).

^b^ Pre-harvest (June -Nov) and post-harvest (Dec-May).

^c^Major diagnoses during admission.

^d^Illnesses within one month before admission as reported by caretaker.

In the younger group, oedematous children had significantly lower prevalence of infection compared to non-oedematous children (25.2% v. 45.1%, p = 0.001). Nevertheless, in the older group the difference in prevalence of infections among oedematous and non-oedematous children was not significant, (5.5% v. 14.3%, p = 0.17). Finally, logistic regression was performed to determine predictors of oedema (Table 4).

**Table 4:** **Factors associated with oedema among 351 children admitted with severe acute malnutrition with odds ratios (OR) and 95**% **confidence intervals (CI)**

	** Simple logistic regression**		**Multiple logistic regression**			
			**Model I**		**Model II**	
	**OR (95% CI)**	**P-value**	**OR (95% CI)**	**P-value**	**OR (95% CI)**	**P-value**
< 5 yr						
Age category, mo						
6-12	Reference		Reference		Reference	
13-24	3.06 (1.46; 6.41)	0.003	3.11 (1.48; 6.55)	0.003	3.04 (1.42;6.53)	0.04
25-36	3.80 (1.77; 8.11)	0.001	3.82 (1.78; 8.18)	0.001	3.67 (1.67;8.02)	0.001
37-59	4.61 (2.00; 10.71)	< 0.001	4.74 (2.04; 11.04)	< 0.001	5.08 (2.10;12.35)	< 0.001
Female sex	1.25 (0.75; 2.10)	0.40	0.76 (0.45; 1.30)	0.35		
Height-for-age Z-score	1.08 (0.93; 1.26)	0.29	1.15 (0.98; 1.35)	0.09		
Admission season^a^						
Pre-harvest	Reference					
Post-harvest	1.43 (0.87; 2.36)	0.16	1.28 (0.76; 2.14)	0.35		
Co-infection b						
TB	0.24 (0.07; 0.78)	0.02	0.16 (0.04; 0.55)	0.004	0.20 (0.06; 0.70)	0.01
Pneumonia	0.44 (0.25; 0.80)	0.007	0.47 (0.22; 0.76)	0.02		
Diarrhea	0.41 (0.22; 0.74)	0.003	0.41 (0.23; 0.81)	0.004	0.40 (0.21; 0.73)	0.003
Malaria	1.92 (0.38; 9.71)	0.43	2.0 (0.37; 10.30)	0.42		
HIV status						
Negative	Reference					
Positive	0.12 (0.01; 1.08)	0.06	0.14 (0.02;1.27)	0.08		
Unknown	1.03 (0.60; 1.79)	0.91	1.15(0.66; 2.02)	0.62		
Reported illness^c^						
Fever	0.68 (0.39; 1.20)	0.18	0.73 (0.41; 1.30)	0.28		
Diarrhea	1.4 (0.74; 2.08)	0.42	1.25 (0.73; 2.14)	0.41		
Cough	0.44 (0.26; 0.73)	0.002	0.48 (0.28; 0.82)	0.007		
Measles	0.48 (0.17; 1.32)	0.15	0.61 (0.21; 1.75)	0.35		
≥ 5 yr						
Age category, mo						
60-95	Reference		Reference			
96-168	0.37 (0.15; 0.91)	0.03	0.39 (0.16; 0.99)	0.05	0.34 (0.13; 0.88)	0.03
Female sex	0.47 (0.20; 1.11)	0.09				
Height-for-age Z-score	0.83 (0.62; 1.12)	0.23	0.83 (0.60; 1.15)	0.27		
Admission season						
Pre-harvest	Reference					
Post-harvest	0.93 (0.39; 2.20)	0.86	1.21 (0.48; 3.05)	0.71		
Co-diagnoses^b^						
TB	0.25 (0.02; 3.2)	0.30	0.20 (0 .01; 2.20)	0.18		
Pneumonia	0.31 (0.04; 2.30)	0.25	0.42 (0 .05; 3.34)	0.42		
Diarrhea	0.15 (0.01; 1.66)	0.12	0.23 (0 .02; 2.18)	0.20		
HIV status						
Negative	Reference					
Positive	0.28 (0.02; 3.20)	0.30	0.38 (0.02; 4.66)	0.45		
Unknown	0.64 (0.25; 1.62)	0.35	0.65 (0.25; 1.71)	0.38		
Reported illness^c^						
Fever	0.52 (0.20; 1.31)	0.17	0.41 (0.15; 1.12)	0.08		
Diarrhea	0.96 (0.40; 2.30)	0.92	0.88 (0.35; 2.18)	0.78		
Cough	0.61 (0.26; 1.42)	0.25	0.60 (0.24; 1.44)	0.25		
Measles	2.67 (0.30; 25.00)	0.38	2.68 (0.27; 27.0)	0.40		

^a^Pre-harvest (June -Nov) and post-harvest (Dec-May).

^b^Major diagnoses during admission.

^c^Illnesses within one month before admission as reported by caretaker.

Model I: adjusted for age and sex.

Model II: adjusted for age, sex, co-infection, admission season and height-for-age Z-score.

The risk of oedema was lower for children 96–168 months of age as compared to 60–95 months (OR = 0.34, 95% CI: 0.13, 0.88). Among the younger children, the odds of oedema was lower in children with TB (OR = 0.20, 95% CI: 0.06, 0.70) or diarrhea, (OR = 0.40, 95% CI: 0.21, 0.73). These factors did not predict oedema in the older group, however.

## Discussion

Most studies on SAM have focused on children under the age of five years. However, as shown in our study, a great proportion of children above the age of 5 were admitted with SAM. Overall, oedematous malnutrition affected around 60% of the children. Additionally, among children under the age of five years a positive relationship was found between age and oedema, whereas in the older children this relationship was reversed. Finally, the risk of oedema was found to be lower in children with infection.

The relationship between age and oedema is a significant finding from our study. There are hardly studies which investigated the age-oedema relationship in older children (> 5 years). Using logistic regression and as shown in Figure 1, the proportion of oedema doubled after infancy with peak at three-five years of age; the odds of oedema was also five times higher at three-five years of age as compared to infants. The odds and proportion of oedema, however, decreased with age after the age of three to five years.

Although the mechanism for this relationship is uncertain, there are some probable explanations. When children start to walk and explore their environment, their risk of acquiring infection or exposure to environmental contaminants is likely to increase [16]. Furthermore, the weaning process and gradual loss of maternally acquired immunity could contribute to increased infection. As a result, this infection or exposure to bacterial endotoxins may increase production of free radicals and oxidative stress [17,18], which may lead to oedema. However, the interaction of immunity and infection and its result might be influenced by age. The requirement for a certain degree of immunocompetence for development of oedema in SAM children was suggested, based on a finding of lower CD4+ percentages in non-oedematous irrespective of their HIV status [19]. Furthermore, a study among Ugandan children showed that half the children hospitalized for severe malnutrition developed oedema after starting ART, although non-oedematous SAM is common in HIV-infected children [20]. So this might be a potential explanation for the higher risk of oedema with increasing age in the first five years. Its subsequent decline might be as a result of better immunity, and as a result lower risk of infection with increasing age.

Infection was found to be lower in oedematous SAM. It seems oedematous SAM is an acute disease usually presenting with shorter duration of illness. Its metabolic dysfunctions resemble that of acute conditions with high case fatality such as toxic shock syndrome and multi-organ failure [21]. Theoretically, this short duration might not be long enough for severe aberration in immunity to develop thus reducing the risk or severity of infection. In hospitals most deaths of SAM children, especially with oedema, are associated with infusion or transfusion [4]. Assessing and managing dehydration/shock in SAM children is also often difficult and incorrect [22].

Younger children with TB were less likely to present with oedema. Macallan [23] showed that TB was associated with wasting, as a result of increased resting energy expenditure and anorexia. Wasting could be due to cytokine induced impairment of amino acids utilization for protein synthesis [24].

Experimental and prospective community studies are recommended to better understand the role of infection-immunity interaction, and effect of age, in the pathogenesis of nutritional oedema [25]. Routine use of antibiotics during treatment of SAM has been questioned [26,27]. A recent trial showed that antibiotics improved recovery and reduced mortality [28]. However, similar evaluation has to be done in areas with low HIV prevalence. Last, in areas where undernutrition is common, older children should be routinely screened for SAM, at least in hospitals, and proper treatment instituted.

Generalization of our finding may be affected by certain limitations of the present study. First, selection bias is an inherent problem of hospital based studies. Hence, the general population of SAM children may not have been well represented. Second, the prevalence of infection might be underestimated due to the absence of detailed and systematic radiological and microbiological investigations to diagnose or exclude infection. Often, diagnosing infection in severely malnourished individuals is difficult and required detailed, and sometimes invasive microbiological investigations. Third, infants less than 6 months were excluded. Although not common, oedema has been documented in this group of children by previous studies [29]. Finally, there might be recall bias in estimating the child’s age. Practically it is impossible to get recorded date of birth as almost all deliveries in rural Ethiopia take place at home [30].

## Conclusion

The following two conclusions can be drawn from the present study. First, proportion of oedematous SAM peaked at three-five years of age. Second, the prevalence of infection was lower among children with oedematous SAM. Although the data are cross-sectional, the relationship suggest that oedema might result from the infection-immunity interaction, which in turn could be influenced by age of the child.

## Competing interests

The authors declare that they have no competing interests.

## Authors’ contributions

TG, PK, KFM, CM and HF were involved in the conception and design of the study. TG, ALH and PK contributed to acquisition of data. TG, PK, KFM, CM and HF contributed to analyses and interpretation of the data. TG was responsible for writing up of the paper while all co-authors reviewed the draft manuscript. All authors read and approved the final manuscript.

## Pre-publication history

The pre-publication history for this paper can be accessed here:

http://www.biomedcentral.com/1471-2431/13/204/prepub
